# Posterior Correction and Fusion Using a 4D Anatomical Spinal Reconstruction Technique Improves Postural Stability Under the Eye-Closed Condition in Patients with Adolescent Idiopathic Scoliosis

**DOI:** 10.3390/jcm13216366

**Published:** 2024-10-24

**Authors:** Satoshi Osuka, Hideki Sudo, Katsuhisa Yamada, Hiroyuki Tachi, Akira Fukushima, Hiroki Mani, Kentaro Watanabe, Fuma Sentoku, Takeshi Chiba, Hiroaki Hori, Norimasa Iwasaki, Masahiko Mukaino, Harukazu Tohyama

**Affiliations:** 1 Faculty of Health Sciences, Hokkaido University, Sapporo 060-0812, Japan; pt-osuka@huhp.hokudai.ac.jp (S.O.); pt-watanabe@huhp.hokudai.ac.jp (K.W.); pt-sentoku@huhp.hokudai.ac.jp (F.S.); pt-chiba@med.hokudai.ac.jp (T.C.); tohyama@med.hokudai.ac.jp (H.T.); 2 Department of Rehabilitation, Hokkaido University Hospital, Sapporo 060-8648, Japan; pt-horihiro@huhp.hokudai.ac.jp; 3 Department of Orthopaedic Surgery, Hokkaido University Hospital, Sapporo 060-8638, Japan; yka2q@pop.med.hokudai.ac.jp (K.Y.); hitachi198885@gmail.com (H.T.); 621akira.fuku@gmail.com (A.F.); niwasaki@med.hokudai.ac.jp (N.I.); 4 Faculty of Welfare and Health Science, Oita University, Oita 870-1192, Japan; mani-hiroki@oita-u.ac.jp; 5 Department of Rehabilitation Medicine, Hokkaido University Hospital, Sapporo 060-8648, Japan; mukaino@huhp.hokudai.ac.jp

**Keywords:** adolescent idiopathic scoliosis, anatomical spinal reconstruction, prospective study, time-to-boundary, center of pressure, force plate

## Abstract

**Background**: Patients with adolescent idiopathic scoliosis (AIS) has been reported to exhibit impaired postural stability. Posterior correction and fusion using four-dimensional (4D) anatomical spinal reconstruction techniques may improve postural stability to correct the spine for optimal anatomical alignment. This prospective study aimed to determine the effect of posterior correction and fusion using a 4D anatomical spinal reconstruction technique on postural stability in the eye-open and eye-closed standing position in patients with thoracic AIS. **Methods**: Thirty-three patients with AIS, excluding those with Lenke type 5C AIS, participated in the study. The mean and standard deviation of the minimum values of the time-to-boundary (TTB) were determined. All patients were asked to perform the quiet standing position under the eye-open and eye-closed condition on a force plate preoperatively and at 1 week and 2 years postoperatively. The TTB value was calculated from the velocity and distance to the foot boundary of the acquired center-of-pressure data. **Results**: Under the eye-closed condition, the mean and standard deviation of the minimum TTB were significantly higher at 2 years postoperatively than preoperatively and at 1 week postoperatively. The mean and standard deviation of the minimum TTB values were significantly lower at 1 week postoperatively than preoperatively. **Conclusions**: The results of this study suggest that surgery using the 4D anatomical spinal reconstruction technique reduces postural stability immediately after surgery; however, it improves postural stability at 2 years compared to the preoperative values.

## 1. Introduction

Adolescent idiopathic scoliosis (AIS) is a common spinal deformity characterized by coronal curvature of the spine with axial rotation and often reduced thoracic kyphosis [1]. Previous studies have indicated that individuals with AIS have poorer postural stability than those without AIS [2,3,4,5,6]. Surgical intervention may be required for patients with AIS with a Cobb angle greater than 40°, and correction of the spinal deformity may alter postural stability in these patients.

The surgical treatment of AIS aims to correct spinal deformities in three dimensions [7,8]. It is particularly important to achieve thoracic kyphosis in the sagittal plane in patients with AIS, especially in those with main-thoracic-curve AIS, because the thoracic kyphosis angle is small and contributes to sagittal imbalance. Sudo et al. developed a four-dimensional (4D) anatomical spine reconstruction technique, which uses spatiotemporal deformation prediction to accurately calculate the postoperative thoracic kyphosis apex, resulting in the correct anatomical curvature of the spine [9]. During the surgical procedure, two pre-bent notch-free rods are shaped to resemble the postoperative spine with the expected apex in the thoracic region between the T6 and T8 vertebrae [10,11]. These pre-bent cobalt chromium rods are identically bent to guide postoperative anatomical thoracic kyphosis without reference to the intraoperative coronal alignment of the AIS deformity [12]. Posterior corrective fixation using the 4D anatomical spinal reconstruction technique to improve radiographic data and the Scoliosis Research Society questionnaire scores have been reported [10,13]. Additionally, Osuka et al. evaluated the postural stability of patients with AIS under an eye-open, single-leg, standing condition and found that the stability improved when comparing the preoperative and 6-month postoperative outcomes [14]. This improvement may have been due to improved postural control based on altered somatosensory perception after spinal correction. However, only postural control was analyzed under the eye-open condition.

Postural stability is believed to be achieved through the sensory integration of visual, vestibular, and somatosensory information [15,16,17,18]. Rectification of the AIS is anticipated to induce sensory reweighting for the regulation of postural control. Sensory reweighting refers to modifying the roles of the visual, vestibular, and somatosensory systems to maintain postural control in instances where there is a modification to one of the sensory modalities [19]. Following corrective spinal surgery, the somatosensory system may be affected by an altered body shape, which may increase the system’s dependency on vision to maintain postural control in the acute postoperative period owing to altered somatosensory perception. Thus, the previous finding [14] that patients with AIS exhibited improved postural stability under the eye-open, single-leg, standing condition 6-month postoperatively could be because of the visual compensation that may have contributed substantially to the postural stability. To clarify the postoperative changes in the effect of somatosensory stimulation on postural control, it is necessary to examine postural stability under the eye-closed condition with long-term follow-up.

The purpose of this prospective study, with a 2-year follow-up period, was to determine the effects of posterior correction and fusion using the 4D anatomical spinal reconstruction technique on postural stability under the quiet, standing, eye-open and eye-closed conditions in patients with AIS. The changes in eye-open and eye-closed postural control demonstrated the efficacy of the surgery using the 4D anatomical spinal reconstruction technique in patients with AIS. In this study, we hypothesized that postural control would improve under both the eye-open and eye-closed conditions and that the eye-closed condition would produce greater changes than the eye-open condition because somatosensory improvement would occur 2 years postoperatively.

## 2. Materials and Methods

### 2.1. Study Design

The present study was a longitudinal prospective study and conducted according to the principles of the Declaration of Helsinki. All patients were enrolled between 2019 and 2022 and followed up for 2 years postoperatively. Prior to conducting and publishing this study, informed consent was obtained from all participants, their guardians, and parents. This study was authorized by the Institutional Review Board of the authors’ affiliated institution(s).

### 2.2. Participants

**Figure 1:**
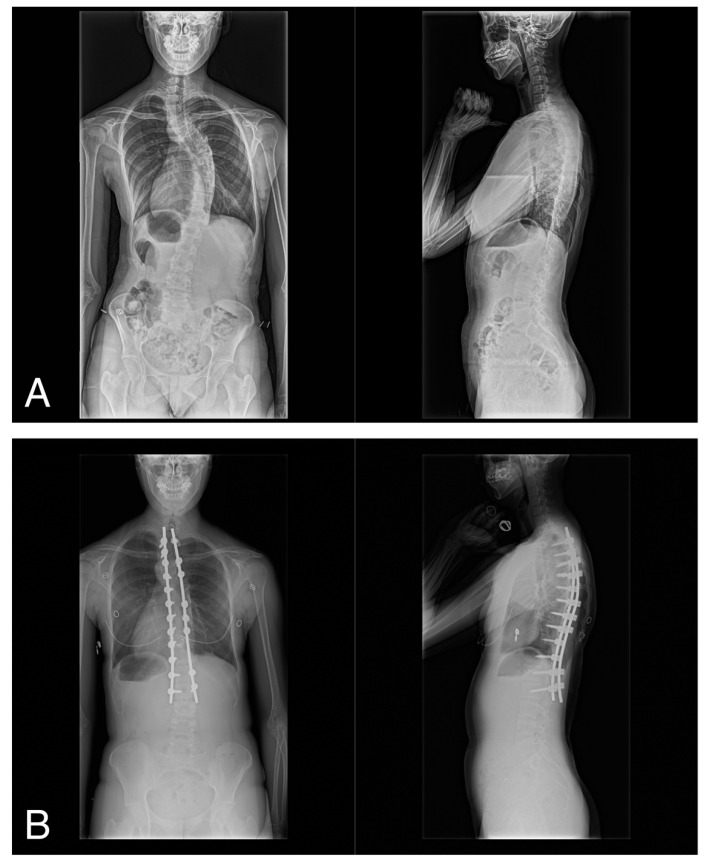
(**A**) Preoperative anteroposterior and lateral views of a 17-year-old woman with Lenke type 1A scoliosis, and (**B**) anteroposterior and lateral radiographs at 2 years postoperatively.

A consecutive group of 33 female patients diagnosed with AIS and categorized into different Lenke types (1A, 1B, 1C, 2A, 3C, and 4C) participated in this study (Table 1). All patients underwent posterior spinal correction and fusion using the 4D anatomical spinal reconstruction technique for AIS correction (Figure 1) [9]. Patients with syndromic, neuromuscular, or congenital scoliosis were excluded from the study. Furthermore, to maintain the focus on the 4D anatomical spinal reconstruction technique, patients with Lenke type 5C AIS, which refers to the main thoracolumbar/lumbar AIS curves, were excluded from the study. The number of participants in the present study was calculated based on the TTB data previously collected during single-leg standing [14]. Sample size calculations showed that a minimum of 19 patients would be required to detect a change in the mean TTB value after surgery (α = 0.05, 1 − β = 0.80).

### 2.3. Postural Stability Evaluation

We used a force plate (MG-1060; Anima Inc., Tokyo, Japan) to assess the center-of-pressure (COP) coordination in both the anteroposterior and lateral directions, while the participants maintained a quiet standing position under the eye-open and eye-closed conditions. The participants were instructed to perform the quiet standing position on the force plate with bare feet for 30 s. This assessment was conducted on three occasions: before surgery, 1 week after surgery, and 2 years after surgery. In all the trials, the stance foot remained consistently positioned at the same spot on the force plate. A total of 3001 time-series COP data points were computed for each trial and sampled at a rate of 100 Hz. Subsequently, a fourth-order low-pass Butterworth filter with a cut-off frequency of 5 Hz was used to refine the COP data.

**Figure 2:**
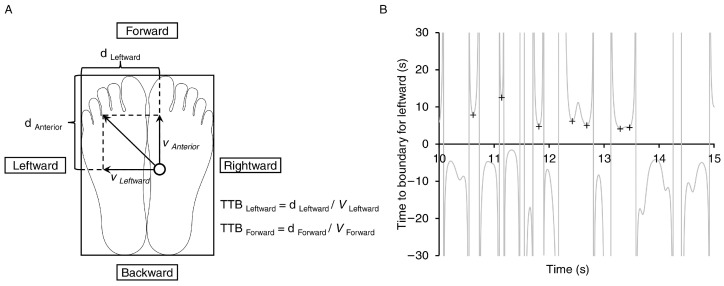
(**A**) The time-to-boundary (TTB) in each direction is calculated by dividing the distance between the center of pressure (open circle) and the imminent boundary of the foot by the corresponding center-of-pressure velocity. Assume that the center of pressure (COP) was shifting toward the forward and leftward directions. Under such circumstances, the TTB is determined by dividing the distance to the forward and leftward limits of the foot by the velocity. (**B**) A typical dataset illustrating the displacement of the center of pressure during testing of eye-closed bilateral standing. The minimum distances to the boundaries in the left region (shown by crosses) are identified at the valleys.

Time-to-boundary (TTB) values were calculated according to previously documented procedures [20,21,22]. To analyze the leftward, rightward, forward, and backward components of the COP, the foot was represented as a rectangular model aligned with its placement on the force plate; for example, to determine the TTB value in the leftward direction, we used the lateral position and velocity of the COP. We calculated the distance between the COP and the lateral edge of the left foot and then divided this distance by the velocity toward the lateral edge of the left foot. This calculation provided the time required for the COP to reach the left foot border while continuing to move in the same direction without changing its velocity (Figure 2A). We then generated a time series of the TTB in the leftward, rightward, forward, and backward directions. For each trial, we identified TTB values in the troughs using the “isocalmin” function in MATLAB 2023b (Figure 2B) [14]. The minima exceeding the mean and two standard deviations were excluded [23]. The mean and standard deviation of the lowest values in the leftward, rightward, forward, and backward directions were calculated. Furthermore, we recorded additional COP metrics, including the maximum velocity in the leftward, rightward, forward, and backward directions of the COP excursions, the range of the COP excursions in the lateral and anteroposterior directions, and the area of the 95% confidence ellipse of the COP movement. The TTB parameters and additional COP metrics were natural logarithms that were transformed to normal distributions [24]. Three trials of the quiet standing eye-open and eye-closed conditions were conducted within a single measurement and the resulting natural logarithm-transformed values were averaged for subsequent analyses.

### 2.4. Radiographic Evaluation

Prior to surgery and at 1 week and 2 years postoperatively, standing long-cassette radiographs were obtained to evaluate various parameters in both the sagittal and coronal planes for all patients. Three spine surgeons with board certifications analyzed all radiographic data. Cobb measurements were obtained for proximal thoracic (PT), main thoracic (MT), and thoracolumbar/lumbar (TL/L) curves. Additionally, we measured the sagittal-plane data, including thoracic kyphosis (T5–T12) and lumbar lordosis (L1–S1) [10].

To assess the global coronal balance, we determined the lateral deviation of the C7 coronal plumb line from the central sacral vertical line (CSVL). The sagittal vertical axis (SVA), which is the exact distance between the C7 plumb line and the posterior upper corner of the S1 superior endplate, was used to measure global sagittal balance. To confirm the regional alignment, the distance between the C7 plumb line and the geometric center of the apical vertebrae was used to measure the apical vertebral translation of the MT curve [10]. Similarly, we identified the distance between the geometric center of the apical vertebrae and CSVL for the TL/L curve [10].

### 2.5. Statistical Analyses

IBM SPSS Statistics 29 (IBM, Chicago, IL, USA) was used to perform the statistical analyses. The study employed a two-way repeated analysis of variance (ANOVA) to assess the disparities in the TTB values and other COP parameters in the preoperative, 1-week-postoperative, and 2-years-postoperative time frames, as well as the differences under the eye-open and eye-closed conditions. For radiographic data, after confirming normality using the Shapiro–Wilk test, one-way repeated ANOVA or the Friedman test was used to compare the preoperative, 1-week-postoperative, and 2-years-postoperative results. A post hoc analysis with Bonferroni correction was performed. The threshold for statistical significance was established at a *p*-value = 0.05. Cohen’s d was used to determine the size of the differences between the groups in the TTB values. Effect sizes were classified as small (d = 0.2), moderate (d = 0.5), or large (d = 0.8) [25]. In addition, the correlation between TTB parameters and radiographic data (Cobb angle, thoracic kyphosis angle, and lumbar lordosis angle) at the final follow-up were examined using the Pearson’s product-moment correlation coefficient. Furthermore, the TTB parameters at the final follow-up were divided into high- and low-value groups using the median value, and intergroup comparisons of radiographic data were conducted using the independent *t*-test or Mann–Whitney U test.

## 3. Results

### 3.1. Postural Stability Parameters

**Figure 3:**
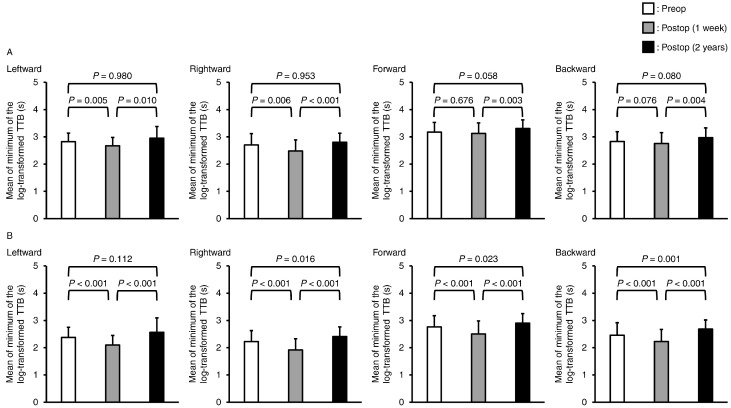
Mean and standard deviation values of the mean minimum of the log-transformed time-to-boundary in leftward, rightward, forward, and backward directions during the quiet standing (**A**) eye-open and (**B**) eye-closed conditions. Because repeated-measures two-way analysis of variance in all comparisons showed significant interactions, a post hoc test with Bonferroni correction was performed.

The minimum mean TTB values are shown in Figure 3. Significant interactions were found in the two-way repeated-measures ANOVA in the leftward, rightward, forward, and backward directions (*p* = 0.004, *p* = 0.008, *p* = 0.003, and *p* < 0.001, respectively). In the post hoc test, the mean of the minimum TTB values in all directions during eye-open standing at 2 years postoperatively was not significantly different compared to preoperatively (leftward: *p* = 0.980, rightward: *p* = 0.953, forward: *p* = 0.058, and backward: *p* = 0.080). In the quiet standing eye-closed condition, the mean minimum TTB values in the rightward, forward, and backward directions were significantly higher at 2 years postoperatively than preoperatively (*p* = 0.016, *p* = 0.023, and *p* = 0.001, respectively). Furthermore, the mean minimum TTB values immediately postoperatively in the leftward and rightward directions in the eye-open standing position were significantly lower than those preoperatively (*p* = 0.005 and *p* = 0.006, respectively). During the quiet standing eye-closed condition, the TTB values in all directions were significantly lower immediately postoperatively than preoperatively (leftward: *p* < 0.001, rightward: *p* < 0.001, forward: *p* < 0.001, and backward: *p* < 0.001). Finally, in the comparison between the immediate postoperative period and 2 years postoperatively, significant differences were found in all directions in the eye-open and eye-closed standing positions. The mean minimum TTB values increased significantly from the immediate postoperative period to 2 years postoperatively. The effect sizes of the mean minimum TTB from the preoperative period to 2 years postoperatively were 0.190–0.334 under the eye-open condition and 0.361–0.546 under the eye-closed condition (Table 2). From the immediate postoperative period to 2 years postoperatively, the effect size was 0.519–0.802 under the eye-open condition and 0.932–1.276 under the eye-closed condition (Table 2).

**Figure 4:**
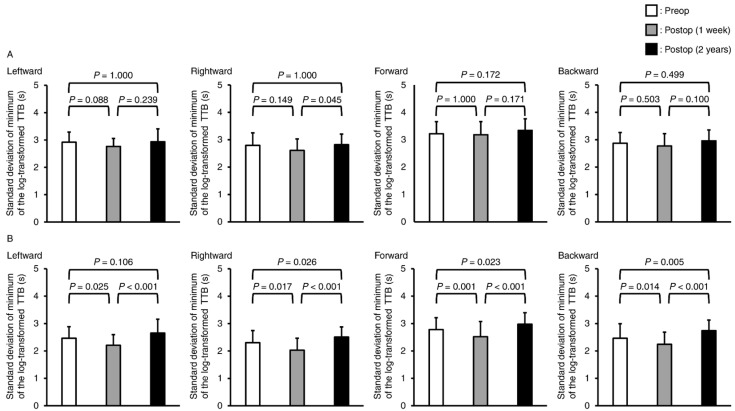
Mean and standard deviation values of the minimum of the log-transformed time-to-boundary in leftward, rightward, forward, and backward directions during the quiet standing (**A**) eye-open and (**B**) eye-closed conditions. Because repeated-measures two-way analysis of variance in all comparisons showed significant interactions, a post hoc test with Bonferroni correction was performed.

Regarding the standard deviation of the minimum TTB, there were also significant interactions in the leftward, rightward, forward, and backward directions in the two-way repeated-measures ANOVA (*p* = 0.004, *p* = 0.006, *p* = 0.011, and *p* = 0.012, respectively) (Figure 4). Under the eye-open condition, significant differences were found only in the comparison between the immediately postoperative and the 2-years-postoperative periods in the rightward direction (*p* = 0.045). In contrast, under the eye-closed condition, the standard deviation of the minimum TTB increased significantly in the rightward, forward, and backward directions from the preoperative to the 2-years-postoperative (*p* = 0.026, *p* = 0.023 and *p* = 0.005, respectively) periods. Furthermore, the standard deviation of the minimum TTB significantly decreased in all directions from preoperatively to immediately postoperatively (leftward: *p* = 0.025, rightward: *p* = 0.017, forward: *p* = 0.001, and backward: *p* = 0.014) and significantly increased from the immediately postoperative to 2-years-postoperative (leftward: *p* < 0.001, rightward: *p* < 0.001, forward: *p* < 0.001, and backward: *p* < 0.001) periods. The effect sizes of the standard deviation of the TTB minimum from the preoperative to the 2-years-postoperative periods were 0.037–0.287 in the eye-open standing position and 0.399–0.584 in the eye-closed standing position (Table 2). Additionally, the effect sizes of the standard deviation of the minimum TTB from the immediately postoperative period to 2 years postoperatively were 0.346–0.517 under the eye-open condition and 0.910–1.190 under the eye-closed condition (Table 2).

The results of the additional COP metrics are listed in Table 3. Under the eye-closed condition, the maximum leftward velocity was significantly lower 2 years postoperatively than preoperatively (*p* = 0.027). Additionally, in the eye-closed condition, although there was a significant increase in the maximum leftward velocity in the immediately postoperative period compared to preoperatively, this value significantly decreased from immediately postoperatively to 2 years postoperatively (*p* < 0.001 and *p* < 0.001, respectively). There was also a significant time main effect on maximal velocity in the rightward and forward directions (rightward: *p* < 0.001, forward: *p* < 0.001), with values at 2 years postoperatively that were significantly lower than preoperatively and immediately postoperatively. In the backward direction, the maximum velocity in the eye-open standing position was significantly lower at 2 years postoperatively than immediately postoperatively (*p* = 0.020). The maximum velocity in the eye-closed standing position was significantly lower 2 years postoperatively than preoperatively and immediately postoperatively (*p* = 0.037 and *p* < 0.001, respectively). Significant effects of time were observed in the range for the lateral and anteroposterior directions (*p* < 0.001 and *p* = 0.021, respectively). In the eye-closed condition, the 95% confidence interval was significantly greater immediately after surgery than at preoperatively and at the final follow-up (*p* = 0.027 and *p* = 0.016, respectively).

### 3.2. Radiographic Parameters

Table 4 summarizes the radiographic parameters in the coronal and sagittal planes. The mean preoperative MT curve was 57.1° (standard deviation, 9.4°). The postoperative radiographs showed a mean MT curve of 12.4° (7.3°). The rate of MT curve correction was 78.7% (10.4%) on average. At the final follow-up, the MT curve, correction rate, and correction angle loss had average values of 14.5° (6.1°), 74.5% (9.9%), and 2.1° (4.7°), respectively. Before surgery, the average PT curve was 26.7° (8.1°). The mean PT curve decreased significantly to 12.2° (5.6°) postoperatively, with an average PT curve correction rate of 53.6% (19.5%). At the final evaluation, the mean values of the PT curve, correction rate, and correction angle loss were 12.2° (6.7°), 54.8% (22.1%), and 0.0° (5.0°), respectively. The mean preoperative TL/L curve was 35.9° (13.7°). The TL/L curve showed a significant decrease to 9.7° (7.2°) postoperatively and remained at 9.2° (6.0°) at the final follow-up (*p* < 0.001 and *p* = 1.000, respectively). Analysis of the sagittal plane demonstrated a significant increase in the average TK from 16.0° (8.4°) before the operation to 25.8° (6.5°) after the operation. Lumbar lordosis showed a significant difference in the ANOVA (*p* = 0.003), and the post hoc test showed a significant difference from the preoperative and 1-week-postoperative periods to the final follow-up (*p* = 0.024 and *p* = 0.009, respectively).

There were significant differences in C7 translation from the CSVL, SVA, and thoracic apical vertebral translation (*p* = 0.047, *p* = 0.009, and *p* < 0.001, respectively). In the post hoc test, there was no significant difference in C7 translation from the CSVL (*p* > 0.005). The SVA changed significantly from the postoperative period to the final follow-up, from −6.6 mm (21.8 mm) to −21.8 mm (24.2 mm) (*p* = 0.011). The mean MT apical vertebral translation showed a significant reduction from 47.3 mm (18.1 mm) before the operation to 11.2 mm (10.2 mm) after the operation and remained at an average of 13.1 mm (8.6 mm) during the final follow-up. There were no significant differences in the TL/L apical vertebral displacement between the measures taken before surgery, after surgery, and during the final follow-up (*p* = 0.216). No patient presented with proximal junctional kyphosis (PJK) at 2 years postoperatively.

Regarding the correlation between TTB values and radiographic data at the final follow-up under the eyes-open condition, significant positive correlations were observed between the Cobb angle of the TL/L curve and the mean of the minimum TTB values in the rightward and backward directions (rightward: *p* = 0.046, *r* = 0.349, and backward: *p* = 0.048, *r* = 0.347, respectively). Additionally, a significant correlation was observed between the Cobb angle of the TL/L curve and the standard deviation of the minimum TTB values in the backward direction (*p* = 0.046, *r* = 0.350). Under the eyes-closed condition, a significant positive correlation was observed between the Cobb angle of the PT curve and the standard deviation of the minimum TTB value in the leftward direction (*p* = 0.042, *r* = 0.356). In addition, in the comparison of the high and low groups in terms of the mean of the minimum TTB values for the leftward and rightward directions at the final follow-up, significant differences were observed in the PT curve, which was significantly larger in the high-value group (*p* = 0.047 and *p* = 0.023, respectively).

## 4. Discussion

This study examined postural stability under the eye-open and eye-closed conditions in patients with AIS and compared them before and after surgery using the 4D anatomical spinal reconstruction technique. Although patients with AIS showed decreased postural control in the immediate postoperative period, the postural control improved 2 years postoperatively compared to that preoperatively in the eye-closed condition. To the best of our knowledge, this is the first study to report that the 4D anatomical spinal reconstruction technique improves postural stability in patients with AIS under the eye-closed condition.

In this study, the mean minimum TTB values during the eye-closed conditions were significantly higher 2 years postoperatively than preoperatively and immediately postoperatively. The standard deviation of the TTB minima showed similar results under the eye-closed condition. The mean value of the minimum TTB represents the time margin to the base of the support boundary, and a small standard deviation indicates a slight postural control strategy [20,26]. These small values were interpreted as indicating a decrease in postural stability. Valles et al. showed that patients with AIS had significantly better postural balance 1 year postoperatively than preoperatively under the quiet standing eye-closed condition [27]. Additionally, Osuka et al. showed that patients with AIS who underwent surgery using a 4D anatomical spinal reconstruction technique had increased TTB parameters during one-leg standing at 6 months after surgery [14]. Thus, the results of this study support those of the previous studies. The TTB values were calculated using the velocity of the COP and distance to the boundary of the base of the support. In the present study, the maximum velocity and range of the COP were significantly lower at the final follow-up than preoperatively, and these changes may have influenced the changes in the TTB parameters.

Interactions between the time frames and eye conditions were observed in the mean and standard deviation of the minimum TTBs. Under the eye-closed condition, although both the mean and standard deviation of the minimum TTB value decreased immediately after surgery, they increased significantly at the final follow-up. Furthermore, for almost all effect sizes, the eye-closed condition showed a greater change than the eye-open condition. Postural stability is believed to be achieved through multisensory integration (i.e., visual, vestibular, and somatosensory integration) [15,16,17,18]. Several authors have proposed that the combination of information from these sensory systems, referred to as sensory reweighting, is actively adjusted to suit the changing environmental conditions and available sensory information [16,28,29,30]. The patients with successfully corrected AIS in this study had an average MT curve correction rate of 78.7%. Such correction may have changed the somatosensory perception, affecting the ability to control posture without visual information, thus lowering the TTB parameters immediately after surgery. Subsequently, our study findings indicated that TTB parameters increased 2 years postoperatively under the eye-closed condition. These results suggest that the improvement in postural control without visual information may be due to the adaptation of appropriate sensory reweighting based on the somatosensory system via the corrected body shape and spinal alignment during the 2 years postoperatively. 

In terms of clinical relevance, it has been reported that patients with AIS have impaired postural stability [2,3,4,5,6]. Postural stability issues are likely to be caused by biomechanical factors such as three-dimensional deformities of the spine. Three-dimensional spinal deformities caused by AIS can lead to asymmetrical trunk muscle activity [31,32] and proprioceptive disorders [33,34]. Correction of spinal alignment is predicted to change muscle activity and proprioception, and improve postural stability. However, there is no consensus regarding the effects of surgery with spinal correction and fusion on postural stability in patients with AIS. Some studies have reported that postural stability decreases or remains unchanged. De Abreu et al. followed up patients with Lenke types 3B and 3C AIS who underwent corrective fixation surgery until 90 days after surgery and reported that the velocity and sway amplitude of the COP were significantly higher than that before surgery [5]. Additionally, St-Georges et al. found that the COP velocity and sway area were not significantly different from the preoperative values from 6 months after surgery in patients with Lenke types 1, 2, 3, 4, 5, and 6 AIS [35]. The difference in the results of this study may be due to the influence of the surgical technique. The 4D anatomical spinal reconstruction technique used in this study is the latest technique for achieving an anatomically correct spine [9]. Many patients with AIS have three-dimensional deformation of the spine, which is not restricted only to the coronal plane, resulting in a reduced thoracic kyphosis angle [36]. In the postoperative period, a progressive decrease in the thoracic kyphotic angle has been reported [37,38,39]. Sudo et al. showed that 4D anatomical spinal reconstruction techniques, including multilevel facetectomy and the use of pre-bent rods based on anatomical spinal alignment, can improve hypokyphosis, and not only coronal but also sagittal-plane spinal alignment [10]. In this study, thoracic kyphosis at 1 week and the final follow-up after surgery was significantly higher than that preoperatively. Furthermore, PJK may manifest as a compensatory mechanism when the TK angle is inadequate after spinal fusion surgery [40]. In the present study, no patient developed PJK, indicating that the administered surgical treatment resulted in sufficient TK angles. In addition, compared with that in previous studies [5,35], the MT Cobb angle in the present study was lower, clearly indicating a better correction. Posterior correction and fusion using the 4D anatomical spinal reconstruction technique may have achieved anatomically appropriate spinal alignment in patients with AIS, which may have led to changes in muscle activity and proprioceptive disorders and contributed to improved postural stability. Immediately after surgery, a temporary decrease in postural stability may occur owing to changes in proprioceptive feedback and muscle activity caused by sudden changes in spinal alignment. Two years later, it is possible that the patients had adapted to the new alignment, and that the proprioceptive sensation and muscle activity had normalized, leading to an improvement in postural stability. Furthermore, in this study, we examined the correlation between TTB values and radiographic data at the final follow-up and compared the radiographic data between the groups with high and low postoperative TTB values. However, no significant results were found for the main sagittal and coronal parameters, and they are thought to have little clinical significance. 

Our study has some limitations. First, the observation period was limited to 2 years. This was a prospective observational study spanning 2 years postoperatively, and its impact over a longer period is unknown. Second, the precise mechanism through which spinal alignment correction improves postural stability remains unclear. To elucidate this, it is necessary to evaluate the proprioception and muscle activity of the trunk and lower extremities during quiet standing. Third, this was a prospective study in which all of the patients underwent 4D anatomical spinal reconstruction. Owing to the lack of a control group, it is difficult to conclude whether the 4D anatomical spinal reconstruction technique has an advantage in terms of postural stability. To demonstrate the effectiveness of this surgery, it is necessary to establish a control group and conduct a randomized controlled trial. Fourth, the patients with AIS in the present study included the 1A, 1B, 1C, 2A, 3C, and 4C classes according to the Lenke classification, and a comparison of the different AIS Lenke classifications was lacking. Therefore, the effects of different scoliosis and fusion segments on postural stability warrant further investigation.

## 5. Conclusions

In conclusion, during quiet standing under the eye-closed condition, patients with AIS who underwent posterior correction and fusion had a significantly higher mean and standard deviation of the minimum TTB values 2 years postoperatively than preoperatively and 1 week postoperatively. These parameters were also significantly lower 1 week postoperatively than preoperatively. Overall, the study findings suggest that although posterior correction and fusion surgery reduces postural stability immediately after surgery, it improves postural stability at 2 years compared to that preoperatively.

## Figures and Tables

**Table 1:** Patient demographic data.

	Mean (Standard Deviation)	Range
Age at surgery, yrs	14.4 (2.2)	11–18
Height at surgery, cm	156.7 (7.6)	140.0–171.0
Weight at surgery, kg	47.3 (8.1)	29.5–62.5
Risser sign	3.6 (1.5)	0–5
Cobb length (upper end to lower end vertebra)	7.7 (0.8)	6–10
Instrumentation length (segments)	10.9 (1.6)	8–14
Operation time, min	251.4 (58.7)	126–402

**Table 2:** Cohen’s d as effect sizes of time-to-boundary parameters.

	Eye-Open Condition			Eye-Closed Condition		
	Preop to Postop (1 week)	Preop to Postop (2 years)	Postop (1 week) to Postop (2 years)	Preop to Postop (1 week)	Preop to Postop (2 years)	Postop (1 week) to Postop (2 years)
Mean of minimum time-to-boundary						
Leftward direction	0.608	0.200	0.681	0.771	0.397	1.002
Rightward direction	0.556	0.190	0.802	0.759	0.483	1.276
Forward direction	0.178	0.334	0.519	0.587	0.361	0.932
Backward direction	0.285	0.305	0.587	0.512	0.546	1.135
Standard deviation of minimum time-to-boundary						
Leftward direction	0.454	0.037	0.409	0.639	0.399	0.970
Rightward direction	0.420	0.056	0.517	0.612	0.496	1.172
Forward direction	0.073	0.287	0.346	0.517	0.458	0.910
Backward direction	0.237	0.228	0.444	0.447	0.584	1.190

**Table 3:** Log-transformed center-of-pressure parameters preoperatively and postoperatively.

	Eye-Open Condition			Eye-Closed Condition			Time-Condition Interaction *p*	Time Main Effect *p*	Condition Main Effect *p*
	Preop	Postop (1 week)	Postop (2 years)	Preop	Postop (1 week)	Postop (2 years)			
Maximum velocity, cm/s									
Leftward direction *^,^**^,^***	1.395 (0.298)	1.539 (0.316) ^a^	1.249 (0.353) ^b^	1.788 (0.344)	2.088 (0.383) ^a^	1.584 (0.409) ^a,b^	<0.001	<0.001	<0.001
Rightward direction *^,^**^,^***	1.431 (0.330)	1.553 (0.342)	1.238 (0.291)	1.783 (0.345)	2.056 (0.411)	1.600 (0.415)	0.078	<0.001	<0.001
Forward direction *^,^**^,^***	1.254 (0.382)	1.363 (0.309)	1.115 (0.273)	1.658 (0.433)	1.853 (0.471)	1.531 (0.334)	0.387	<0.001	<0.001
Backward direction *^,^***	1.241 (0.411)	1.301 (0.325)	1.156 (0.346) ^b^	1.638 (0.415)	1.909 (0.462) ^a^	1.502 (0.350) ^a,b^	<0.001	<0.001	<0.001
Range, cm									
Lateral direction **^,^***	0.828 (0.236)	0.877 (0.284)	0.701 (0.370)	1.144 (0.309)	1.242 (0.340)	0.961 (0.391)	0.083	<0.001	<0.001
Anteroposterior direction *	0.815 (0.329)	0.904 (0.293)	0.797 (0.377)	1.001 (0.317)	1.174 (0.384)	1.018 (0.381)	0.184	0.021	<0.001
95% confidence ellipse area, cm^2^	1.352 (0.541)	1.403 (0.537)	1.214 (0.709)	1.765 (0.627)	1.999 (0.703) ^a^	1.614 (0.697) ^b^	0.007	0.019	<0.001

The values are presented as the mean (standard deviation). ^a^ Significant differences compared to preoperatively in each group (*p* < 0.05). ^b^ Significant differences compared to immediately postoperatively in each condition (*p* < 0.05). * Significant differences compared with preoperatively and immediately postoperatively for the post hoc test for time main effect (*p* < 0.05). ** Significant differences compared with preoperatively and at the final follow-up for the post hoc test for time main effect (*p* < 0.05). *** Significant differences compared with immediately postoperatively and at the final follow-up for the post hoc test for time main effect (*p* < 0.05).

**Table 4:** Radiographic parameters preoperatively and postoperatively.

	Preop	Postop (1 week)	Postop (2 years)	Overall *p*	Post Hoc Test *p*		
					Preop to Postop (1 week)	Preop to Postop (2 years)	Postop (1 week) to Postop (2 years)
Coronal-plane data							
Proximal thoracic curve, degree	26.7 (8.1)	12.2 (5.6)	12.2 (6.7)	<0.001	<0.001	<0.001	1.000
Main thoracic curve, degree	57.1 (9.4)	12.4 (7.3)	14.5 (6.1)	<0.001	<0.001	<0.001	0.329
Thoracolumbar/Lumbar curve, degree	35.9 (13.7)	9.7 (7.2)	9.2 (6.0)	<0.001	<0.001	<0.001	1.000
Sagittal-plane data							
Thoracic kyphosis (T5 to T12), degree	16.0 (8.4)	25.8 (6.5)	26.4 (6.7)	<0.001	<0.001	<0.001	1.000
Lumbar lordosis (L1 to S1), degree	44.3 (13.3)	45.2 (9.4)	51.4 (12.8)	0.003	1.000	0.024	0.009
Balance parameters and translational data							
C7 translation from central sacral vertical line, mm	16.6 (11.3)	15.8 (11.2)	10.0 (9.3)	0.047	1.000	0.058	0.255
Sagittal vertical axis, mm	−13.0 (18.9)	−6.6 (21.8)	−21.8 (24.2)	0.009	0.483	0.257	0.011
Thoracic apical vertebral translation, mm	47.3 (18.1)	11.2 (10.2)	13.1 (8.6)	<0.001	<0.001	<0.001	1.000
Thoracolumbar/Lumbar apical vertebral translation, mm	18.4 (16.7)	12.2 (9.6)	12.5 (8.8)	0.216	-	-	-

The values are presented as the mean (standard deviation).

## Data Availability

The data that support the findings of this study are available from the corresponding author on reasonable request.
